# First proteomic analysis of the role of lysine acetylation in extensive functions in *Solenopsis invicta*

**DOI:** 10.1371/journal.pone.0243787

**Published:** 2020-12-16

**Authors:** Jingwen Ye, Jun Li

**Affiliations:** Guangdong Key Laboratory of Animal Conservation and Resource Utilization, Guangdong Public Laboratory of Wild Animal Conservation and Utilization, Institute of Zoology, Guangdong Academy of Science, Guangzhou, Guangdong Province, The People’s Republic of China; Saint Louis University School of Medicine, UNITED STATES

## Abstract

Lysine acetylation (Kac) plays a critical role in the regulation of many important cellular processes. However, little is known about Kac in *Solenopsis invicta*, which is among the 100 most dangerous invasive species in the world. Kac in *S*. *invicta* was evaluated for the first time in this study. Altogether, 2387 Kac sites were tested in 992 proteins. The prediction of subcellular localization indicated that most identified proteins were located in the cytoplasm, mitochondria, and nucleus. Venom allergen Sol i 2, Sol i 3, and Sol i 4 were found to be located in the extracellular. The enriched Kac site motifs included Kac H, Kac Y, Kac G, Kac F, Kac T, and Kac W. H, Y, F, and W frequently occurred at the +1 position, whereas G, Y, and T frequently occurred at the –1 position. In the cellular component, acetylated proteins were enriched in the cytoplasmic part, mitochondrial matrix, and cytosolic ribosome. Furthermore, 25 pathways were detected to have significant enrichment. Interestingly, arginine and proline metabolism, as well as phagosome, which are related to immunity, involved several Kac proteins. Sequence alignment analyses demonstrated that V-type proton ATPase subunit G, tubulin alpha chain, and arginine kinase, the acetylated lysine residues, were evolutionarily conserved among different ant species. In the investigation of the interaction network, diverse interactions were adjusted by Kac. The results indicated that Kac may play an important role in the sensitization, cellular energy metabolism, immune response, nerve signal transduction, and response to biotic and abiotic stress of *S*. *invicta*. It may be useful to confirm the functions of Kac target proteins for the design of specific and effective drugs to prevent and control this dangerous invasive species.

## Introduction

Post-translational modifications, which include acetylation, succinylation, phosphorylation, ubiquitylation, and so forth [1], monitor protein activity in the great majority of eukaryotes [2]. Lysine acetylation (Kac) varies dynamically with time and is reversible. It is involved in a great deal of biological functions, including the modification of targets, regulation of gene expression, and maintenance of protein stability [3, 4]. A large number of Kac proteins have been observed in an extensive scope of biological processes, including the growth and metabolism of cells, apoptosis, immune response, and autophagy, in numerous species [5–7].

Histone Kac, which participates in a variety of cellular processes, including the activation of transcription, silencing of genes, repairing of DNA, and progression of the cell cycle, is catalyzed by histone acetyltransferases (HATs) as well as histone deacetylases (HDACs), utilizing acetylated coenzyme A as an accessory factor [8, 9]. The balancing behavior of HATs and HDACs is vital for cellular development and function [10]. Genomic analyses of *Trichinella spiralis* have shown that there are many HAT and HDAC orthologs, indicating that Kac may be important for the development and metabolism of *T*. *spiralis* [11]. Further, non-histone Kac plays an important role in crucial regulatory processes, including gene transcription, DNA repair, cell division, signal transduction, autophagy, proteins folding, and metabolism [8]. Residues of Kac have been discovered in a wide range of species, including microbes, plants, insects, and mammals [8, 9]. Weinert et al. [12] reported a high degree of conservation of Kac in *Drosophila melanogaster*. Nie et al. [9] demonstrated the acetylome of *Bombyx mori*. Hu et al. [1] found that viral proteins were acetylated by HAT of the host (*B*. *mori*). However, Kac in Hymenoptera insects has received little attention.

*Solenopsis invicta* (Hymenoptera: Formicidae) is among the 100 most dangerous invasive species in the world. In September, 2004, *S*. *invicta* was found for the first time in Wuchuan, Guangdong Province, in mainland China. It has proven harmful to public safety and health, the environment, and the production of agriculture and forestry [13]. *S*. *invicta* is generally found near water resources, including parks, docks, schools, and public green spaces [14]. *S*. *invicta* fiercely attacks anything that disturbs its nest, even damaging wires in electrical boxes [15].

A synthetic method that incorporated affinity enrichment and Liquid chromatography-tandem mass spectrometry (LC/MS/MS) was used to test the dynamic variation in the global acetylome of *S*. *invicta*. This constitutes the first proteomic analysis of Kac in *S*. *invicta*. Altogether, 2387 acetylated sites were tested in 992 Kac proteins. These acetylated proteins participated in a variety of cellular processes and biological functions, particularly in the cytoplasm. It has been found that a wide range of functions are monitored by Kac in *S*. *invicta*, and our data represent abundant information that can be used for future functional analyses of reversible Kac in *S*. *invicta* and other Hymenoptera insects.

## Methods

### Protein extraction and detection of Kac proteins

*S*. *invicta* was collected from wasteland in Huangpu, Guangzhou, China. The workers were abraded in liquid nitrogen and diverted to a 5 -mL centrifuge tube. The powder was dissolved in dissolution buffer (3 μM trichostatin A, 50 mM nicotinamide, 1% protease inhibitor cocktail, 10 mM dithiothreitol, Tris pH 8.0, and 0.1 M ammonium sulfate-saturated methanol) on ice. Trichostatin A and nicotinamide are histone deacetylases inhibitors. After centrifugation at 5500 g at 4°C for 10 min, the liquid supernatant was gathered, and the concentration of protein was confirmed with a BCA kit, in accordance with the manufacturer’s instructions.

### Trypsin digestion

For the following steps, 1.5 mg protein was used. Reduction of proteins with 5 mM dithiothreitol was carried out for 30 min at 56°C during first digestion, and the alkylation of proteins with 11 mM iodoacetamide was implemented for 15 min at room temperature in darkness. Then, 100 mM TEAB was added to thin the protein so that the final concentration of urea was less than 2M, and overnight digestion with trypsin was performed at a mass ratio of 1: 50 trypsin: protein. For the second digestion, trypsin was added at a mass ratio of 1:100 trypsin:protein and digested for 4 h.

### Affinity enrichment and LC-MS/MS analyses

To concentrate the acetylated peptides, the peptides of trypsin were lysed in NETN buffer (0.5% NP-40, 50 mM Tris-HCl, 100 mM NaCl, 1 mM EDTA, pH 8.0). Next, they were incubated with pre-washed beads of anti-acetyl lysine antibody and softly shaken at 4°C overnight. Then, the peptides were eluted from the beads, which were scoured four times with NETN buffer and twice with ddH_2_O, with 0.1% trifluoroacetic acid, vacuum- dried in a Speed Vac, and desalted with C18 Zip Tips (Millipore), in accordance with the manufacturer’s instructions. The synthetic peptides were tested using tandem mass spectrometry (MS/MS) in Q Exactive TM plus (Thermo), which was connected online to the UPLC. Using the Max Quant search engine (v.1.5.2.8), the tandem mass spectra were queried against the UniProt *S*. *invicta* database. The parameters were as follows: trypsin/P was appointed as a lyase, in which up to four lacking cleavages, five modifications per peptide, and five charges were permitted. The mass tolerance of the precursor ions was set as 5 ppm, and that of the fragment ions was set to 0.02 Da. The error-detection rates of protein, peptide, and modification sites were adjusted to < 1%, and the minimum score of modified peptides was set to >40.

### Bioinformatics analyses

Gene ontology (GO) annotation of Kac proteins was extracted from the UniProt-GOA database, following Chen et al. [16]. First, identified protein IDs were converted to UniProt IDs and then mapped to GO IDs by protein ID. For identified proteins that were not annotated by the UniProt-GOA database, the InterProScan software was used to obtain its GO functional based on the protein sequence alignment method. Then, proteins were annotated in terms of three aspects: biological process, cellular component, and molecular function. Domain annotation was carried out using InterProScan on the database of the InterPro domain. Central to the database are diagnostic models, known as signatures, against which protein sequences can be searched to determine their potential functions. InterPro is useful for large-scale analyses of whole genomes and meta-genomes, as well as for characterizing individual protein sequences. The Kac protein pathways were annotated using the database of the Kyoto Encyclopedia of Genes and Genomes (KEGG). First, using KEGG’s online service tool KAAS, the annotated protein’s KEGG database description was taken. Then, the annotation result was mapped in the KEGG pathway database using the KEGG mapper online service tool. Wolfpsort was applied to predict subcellular localization. Wolfpsort is an updated version of PSORT/PSORT II, which can predict eukaryotic sequences. Software MoMo (motif-x algorithm) was used to evaluate the motifs of amino acid sequences in appraised proteins. The least number of appearances was set as 20. Fisher’s exact test was employed to test the GO, KEGG pathway, and domain enrichments, and they were considered significant for corrected p-values < 0.05. All identified acetylated protein sequences were blasted to *Acromyrmex echinatior*, and protein–protein interactions were searched using version 10.5 of the STRING database. All interactions with a confidence level ≥ 0.7 (high confidence) were acquired. The interaction network derived from STRING was visualized in the R package networkD3. The mass spectrometry proteomics data have been deposited to the ProteomeXchange Consortium via the PRIDE [1] partner repository with the dataset identifier PXD019033.

### Western blotting

Western blot evaluation was performed with a pan anti-acetyl lysine antibody (PTM Biolabs, Hangzhou, China) diluted at a 1:1000. The supernatants were gathered and splited by using 12% SDS polyacrylamide gel. Next, isolated proteins were electrically transferred to an NC membrane (BioRad, 0.2 μm) by the wet transfer system. After that, the membrane was sealed for 90 min with 5% BSA. The membrane was washed and incubated with the pan anti-acetyl lysine antibody, and then Kac proteins were disclosed. A horseradish peroxidase-labeled goat anti-mouse IgG antibody (1:5000) was employed for second antibody.

## Results

### Identification and analysis of Kac proteins and sites in *S*. *invicta*

**Fig 1:**
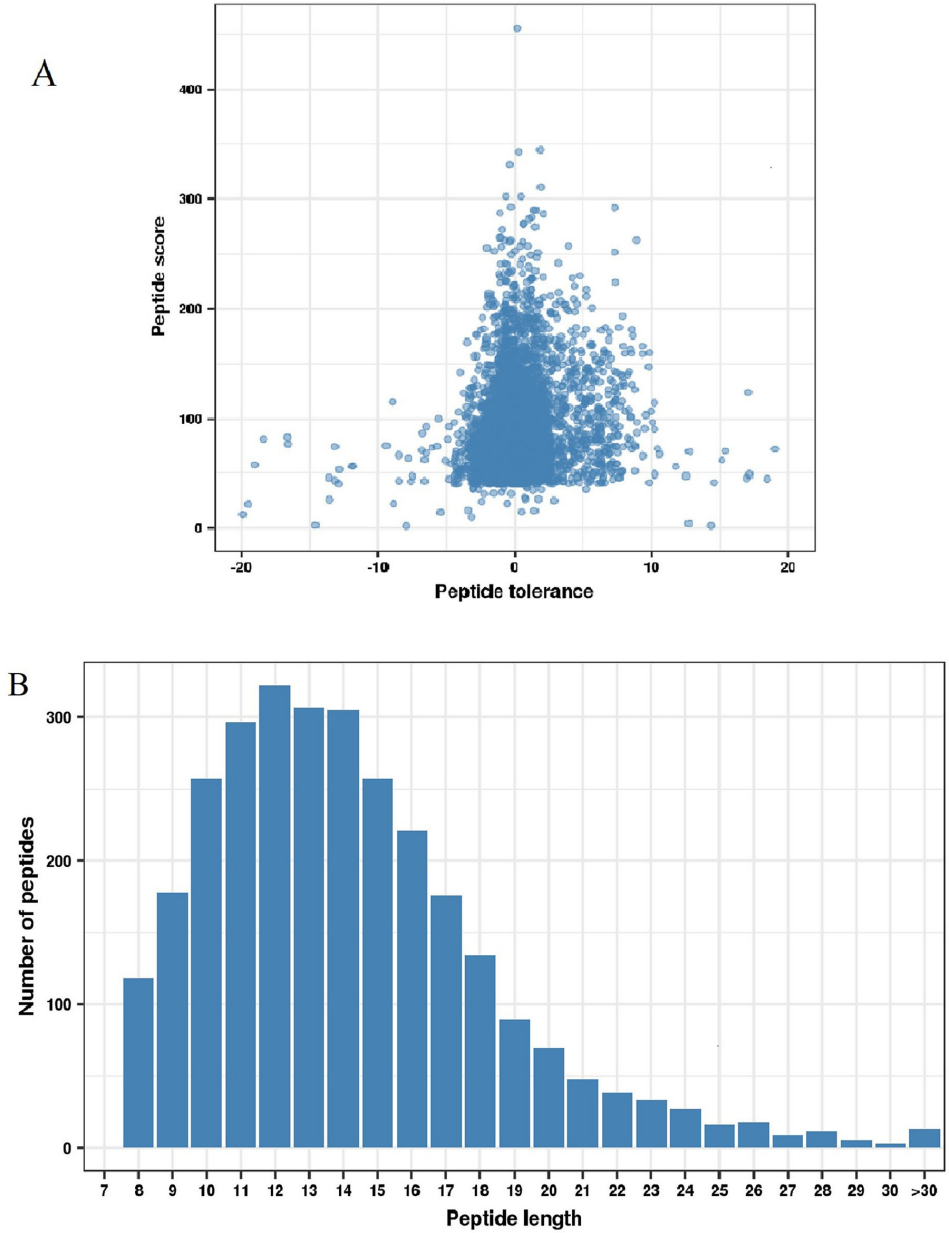
Identification of lysine acetylation proteins in *Solenopsis invicta*. (A) Mass error distribution of all identified peptides. (B) Distribution of acetylated peptide length.

**Fig 2:**
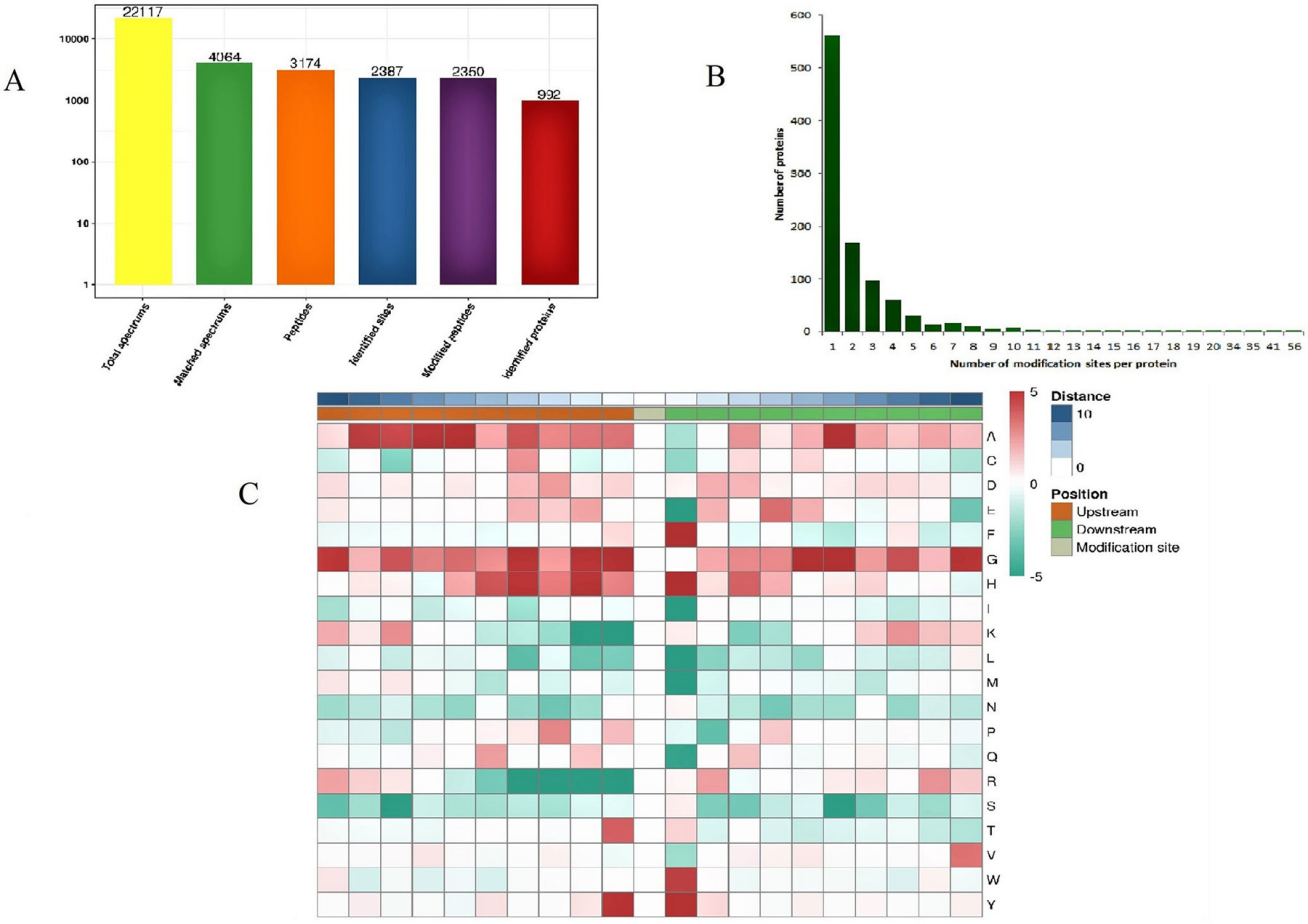
Identification of lysine acetylation sites in *Solenopsis invicta*. (A) Basic statistical figure for MS results. (B) Numbers of acetylated sites per protein. (C) Motif enrichment heat map of upstream and downstream amino acids for all identified Kac sites. Red indicates amino acids that are significantly enriched near the Kac site, and green indicates amino acids that are significantly reduced near the Kac site.

The average peptide value was 94.73, and the distribution of mass errors was close to zero (Fig 1A). All Kac peptides were between 8 and 33 amino acids in length, mostly between 8 and 19 amino acids (Fig 1B). In all, 2387 acetylation sites were tested in 992 proteins (Fig 2A). Among the Kac proteins, 562 proteins (56.65%) had one Kac site, and 104 proteins (10.48%) contained five or more Kac sites. There were 10 proteins (1.01%) that contained > 15 Kac sites. On average, there were 2.41 sites per protein (Fig 2B). The peptides were observed to contain 20 amino acid residues, ranging from position –10 to +10 surrounding acetylated lysine. The results of motif evaluation revealed an enrichment of alkaline amino acids (H), aromatic amino acids (F, Y, and W), fatty amino acids (G), and hydroxyl amino acids (T). H, Y, F, and W frequently occurred at the +1 position, whereas G, Y, and T frequently occurred at the –1 position. Among these, Y was enriched both at the +1 and –1 position (Fig 2C).

### Gene Ontology (GO) classification

**Fig 3:**
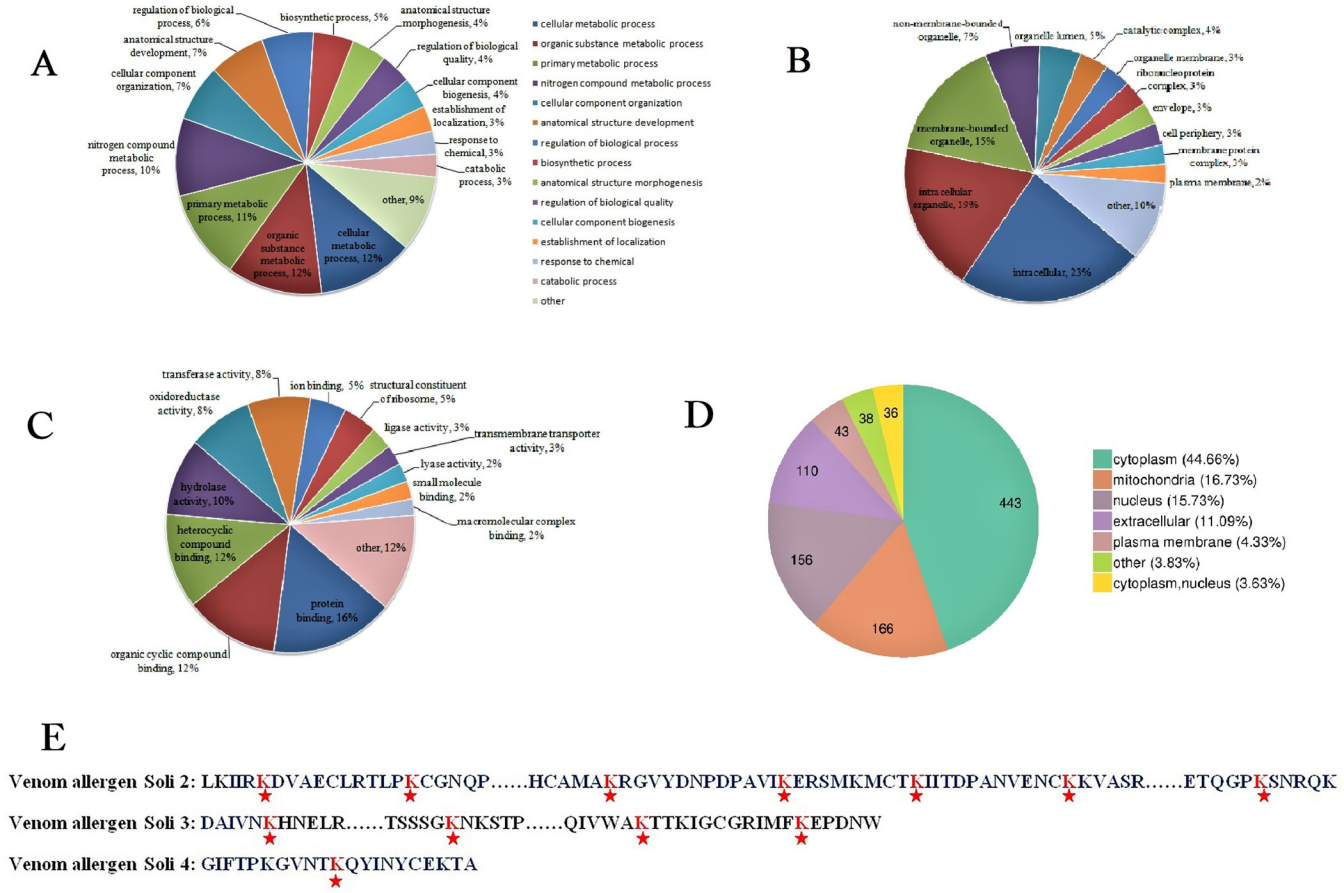
Gene Ontology (GO) classification and subcellular location prediction of lysine acetylation proteins in *Solenopsis invicta*. The GO categories indicated biological process, cellular component, and molecular function. (A) GO classification based on biological process. (B) GO classification based on cellular component. (C) GO classification based on molecular function. (D) Subcellular localization of proteins corresponding to Kac sites. (E) Kac sites of venom allergen Sol i 2, Sol i 3, and Sol i 4 located in the extracellular.

A functional classification of the identified proteins was carried out to clarify the possible roles of Kac. Cellular metabolic process, organic substance metabolic process, primary metabolic process and nitrogen compound metabolic process accounted for a large proportion of functions (Fig 3A). The top three GO terms under the cellular component contained intracellular, intracellular organelle, and membrane-bounded organelle (Fig 3B). In the classification of molecular functions, the proteins were mainly involved in protein binding, organic cyclic compound binding, and heterocyclic compound binding (Fig 3C).

### Subcellular location prediction

Predictions of subcellular location indicated that most proteins were located in the cytoplasm (44.66%), mitochondria (16.73%), and nucleus (15.73%). Moreover, a lot of acetylated proteins have been established to be extracellular (11.09%) or plasma membranous (4.33%) proteins (Fig 3D). Venom allergen Sol i 2, Sol i 3, and Sol i 4 (7, 4, and 1 Kac sites, respectively) were found to be located in the extracellular (Fig 3E).

### GO, KEGG, and domain enrichments

**Fig 4:**
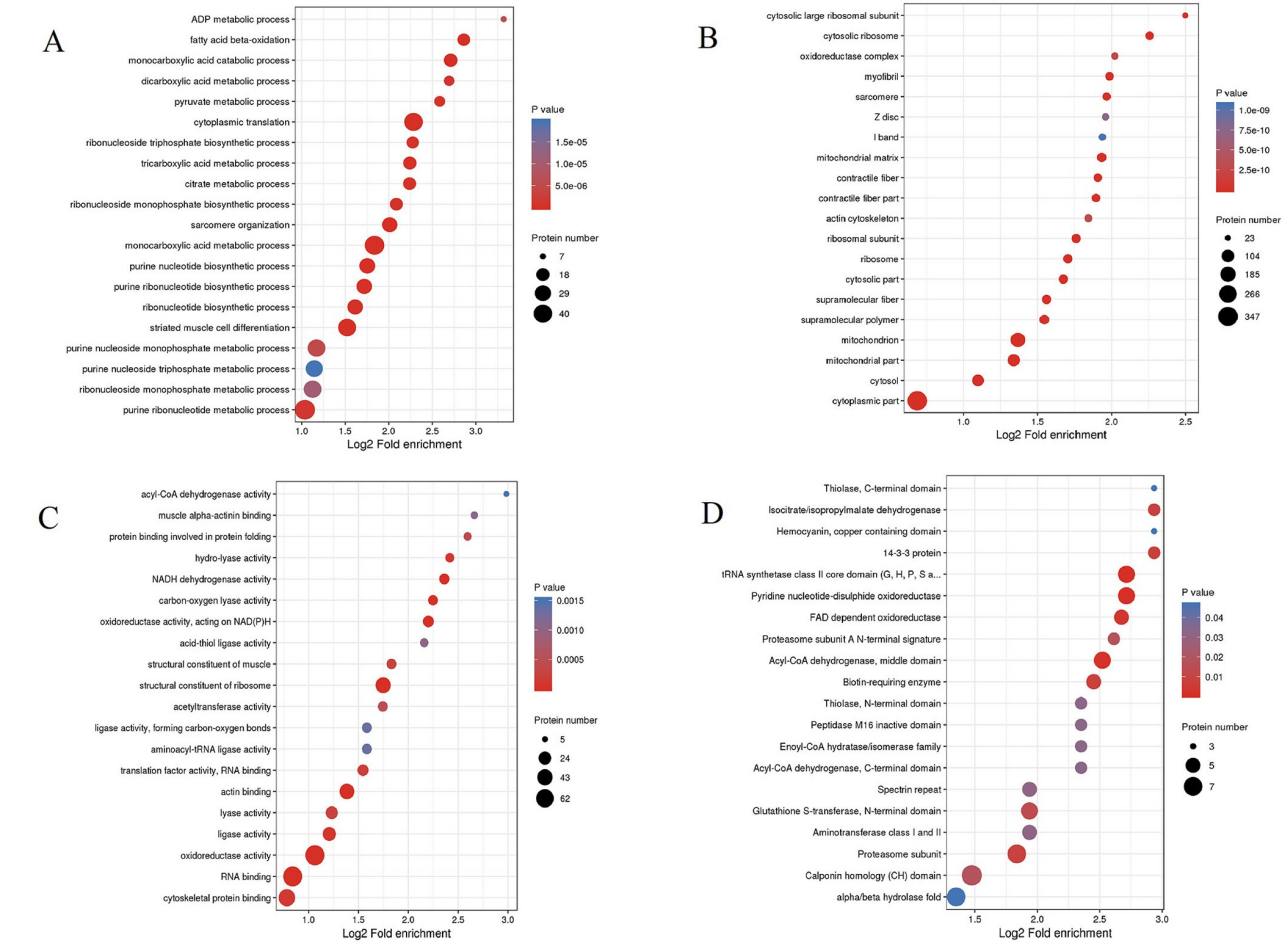
Gene Ontology (GO) and domain enrichment of proteins corresponding to lysine acetylation sites in *Solenopsis invicta*. (A) GO enrichment bubble plot of proteins in biological processes. (B) GO enrichment bubble plot of proteins in cellular components. (C) GO enrichment bubble plot of proteins in molecular functions. (D) Protein domain enrichment bubble plot of proteins.

GO enrichment was performed to better understand the three protein types of Kac. GO enrichment of biological process revealed significant protein enrichment, which were related to cytoplasmic translation, monocarboxylic acid metabolic process, and monocarboxylic acid catabolic process (*p* < 0.0001; Fig 4A). In the cellular component, the results indicated that acetylated proteins were enriched in cytoplasmic part, mitochondrial matrix, cytosolic ribosome, and others (*p* < 0.0001; Fig 4B). The results also showed that proteins related to the structural constituents of ribosome, to oxidoreductase activity, and to actin binding were significantly enriched (*p* < 0.0001; Fig 4C).

Domain enrichment analyses found that 25 protein domains, including pyridine nucleotide-disulphideoxido reductase, the tRNA synthetase class II core domain (G, H, P, S, and T), Acyl-CoA dehydrogenase, the middle domain, and FAD dependent oxidoreductase, were significantly enriched substrates (Fig 4D). Moreover, several Kac proteins containing different domains, including the tRNA synthetase class II core domain (G, H, P, S, and T), calponin homology (CH) domain, spectrin repeat, proteasome subunit, and proteasome subunit A N-terminal signature, had a range of numbers of acetylation sites (S1 Table).

**Fig 5:**
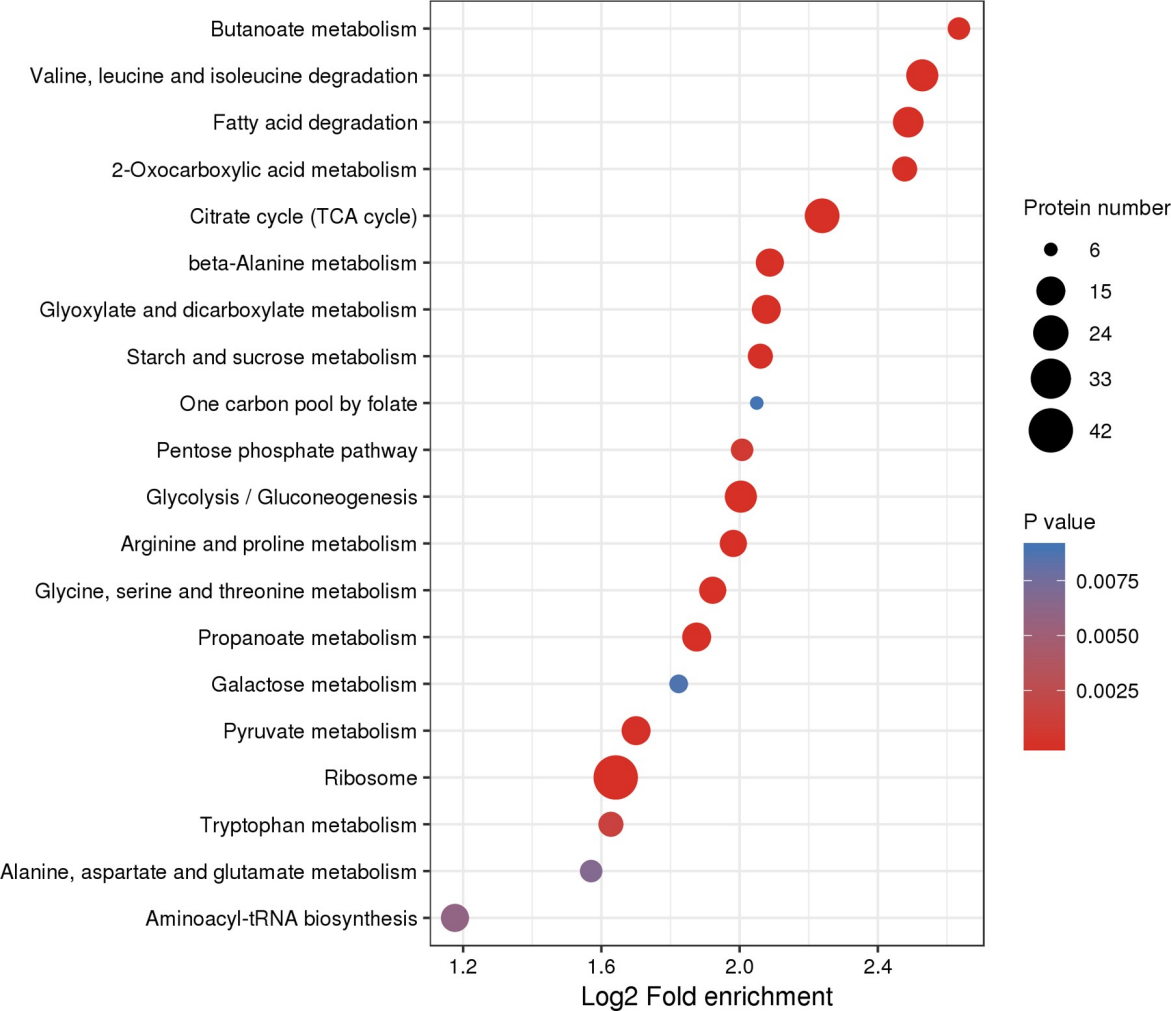
Bubble plot of KEGG pathway enrichment of proteins corresponding to lysine acetylation sites in *Solenopsis invicta*.

**Fig 6:**
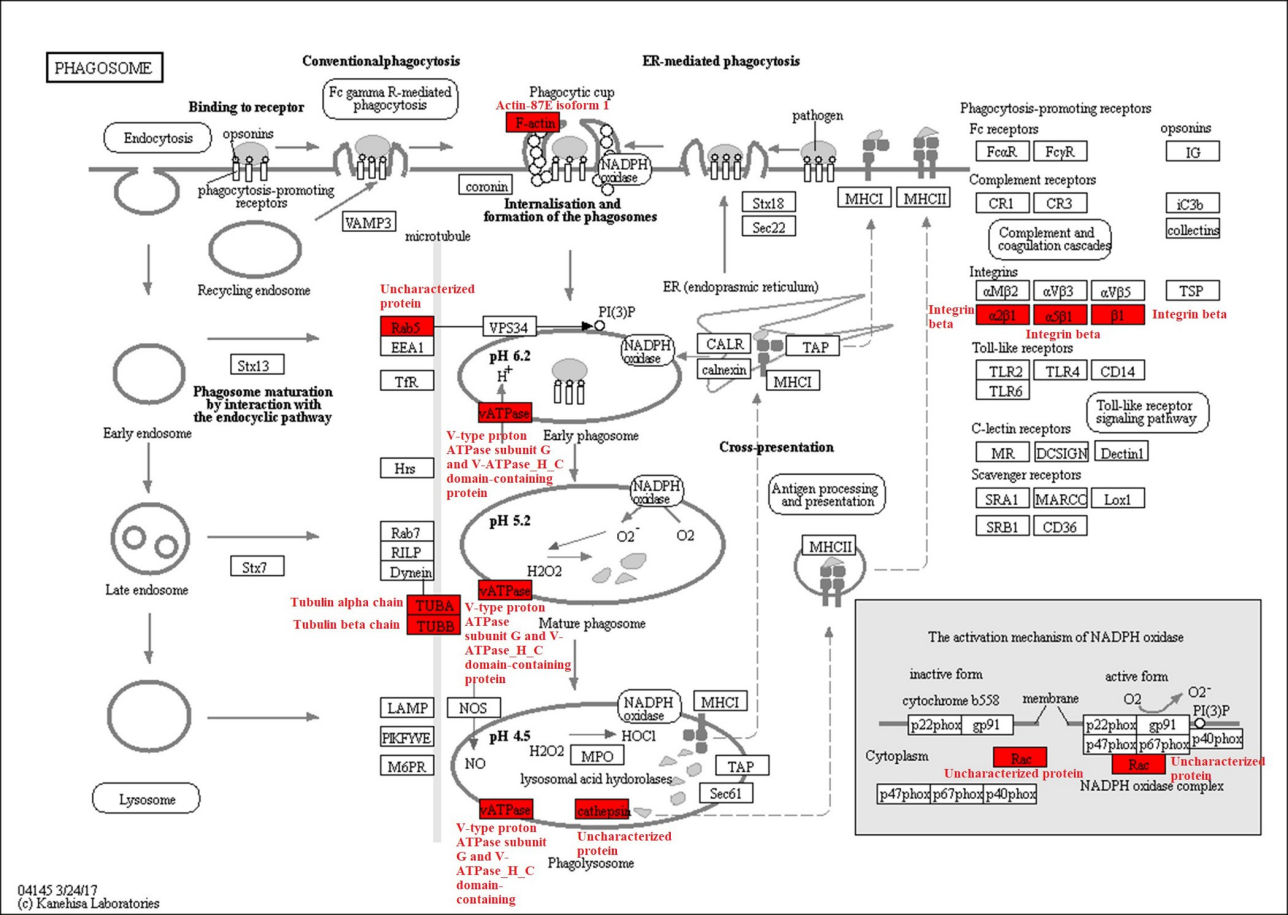
Significantly enriched KEGG pathway in phagosome of *Solenopsis invicta*. Lysine acetylation proteins are marked in red.

**Fig 7:**
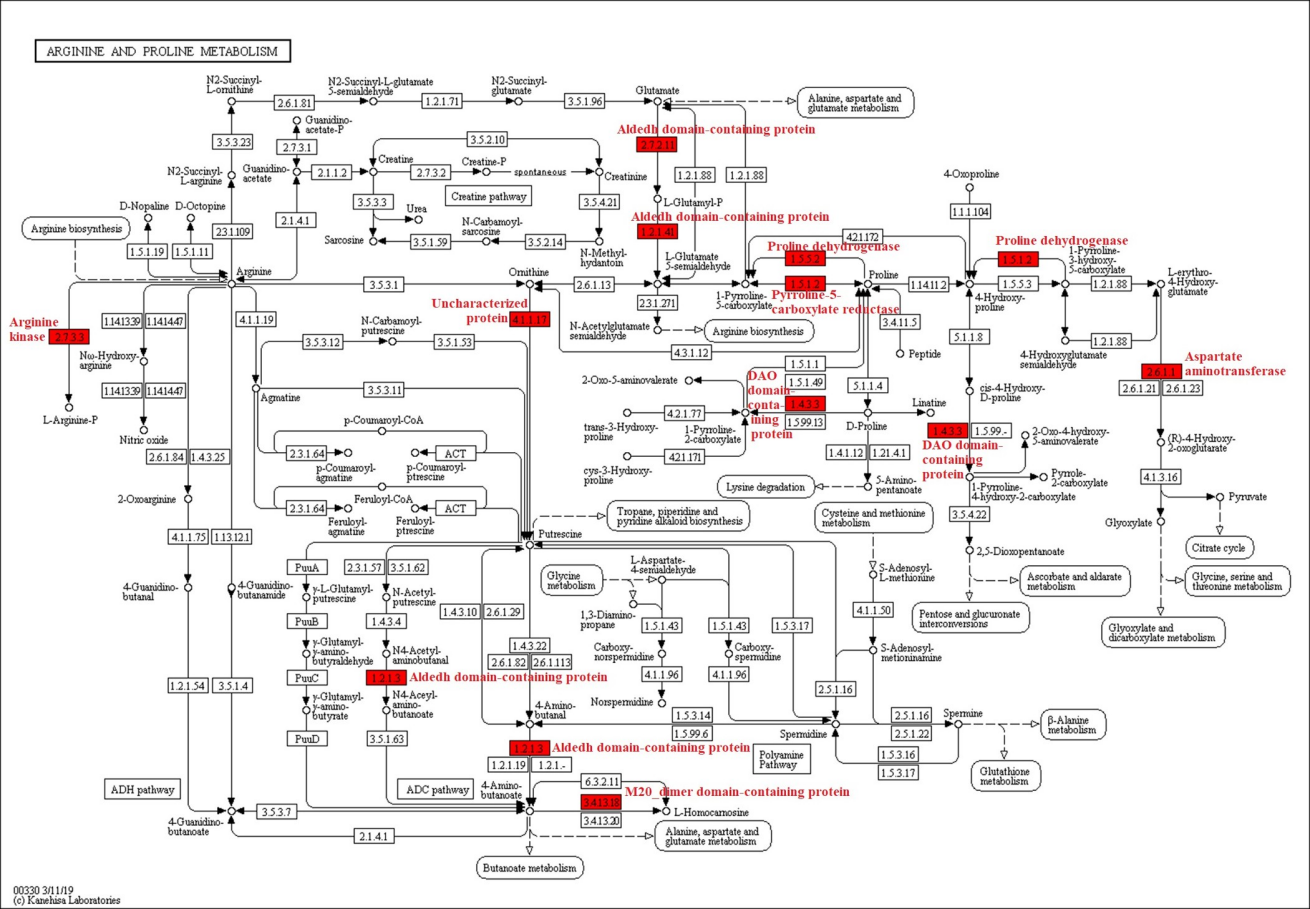
Significantly enriched KEGG pathway in arginine and proline metabolism of *Solenopsis invicta*. Lysine acetylation proteins are marked in red.

**Fig 8:**
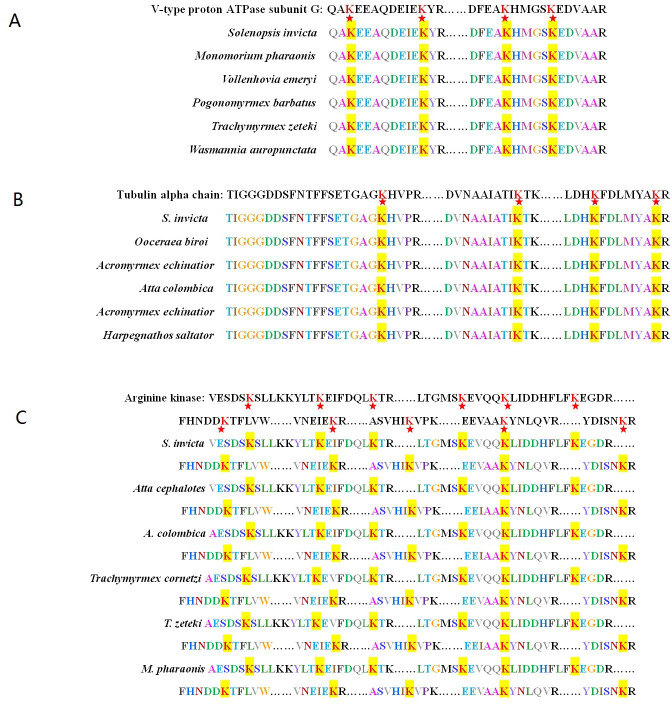
V-type proton ATPase subunit G and tubulin alpha chain in *Solenopsis invicta* were conserved among various ant species. The red star denotes the conserved lysine residues, and these results were analyzed by DNASTAR software. (A) V-type proton ATPase subunit G. (B) Tubulin alpha chain. (C) arginine kinase.

In the KEGG pathway-enrichment analyses, 25 pathways were detected to be significantly enriched, including ribosome, valine, leucine and isoleucine degradation, citrate cycle (TCA cycle), fatty acid degradation, glycolysis/gluconeogenesis, and others (*p* < 0.0001; Fig 5). The phagosome contained several Kac proteins, for example, the V-type proton ATPase subunit G, tubulin alpha chain, V-ATPase_H_C domain-containing protein, Actin-87E isoform 1, and tubulin beta chain (*p* < 0.05; Fig 6). In addition, the arginine and proline metabolism also included many Kac proteins, including the aldedh domain-containing protein, pyrroline-5-carboxylate reductase, aspartate aminotransferase, M20_dimer domain-containing protein, proline dehydrogenase, and arginine kinase (*p* < 0.0001; Fig 7). Sequence alignment analyses demonstrated that V-type proton ATPase subunit G, tubulin alpha chain, and arginine kinase, the acetylated lysine residues, were evolutionarily conserved among different ant species (Fig 8A–8C).

### Protein-Protein Interaction (PPI) networks

**Fig 9:**
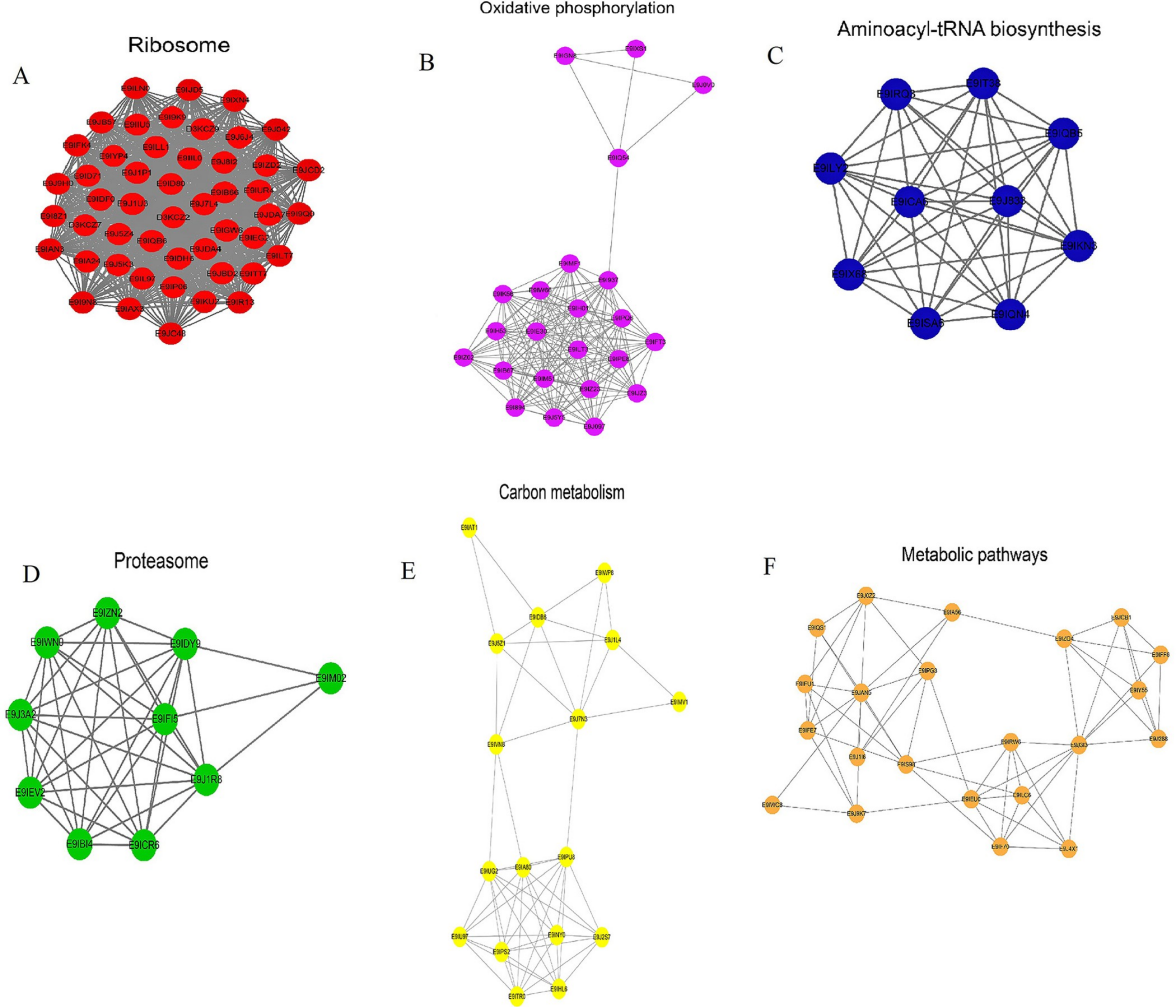
Protein–Protein Interaction (PPI) networks of proteins corresponding to lysine acetylation sites in *Solenopsis invicta*. (A) PPI network related to ribosome. (B) PPI network related to oxidative phosphorylation. (C) PPI network related to aminoacyl-tRNA biosynthesis. (D) PPI network related to proteasome. (E) PPI network related to carbon metabolism. (F) PPI network related to metabolic pathways.

PPI networks were assembled to further investigate the processes adjusted by Kac in *S*. *invicta*. A PPI network was constructed of 220 acetylated proteins that were identified as nodes and connected with each other. Most of the proteins in the PPI network contained numerous Kac sites. The six most interconnected interaction clusters, namely, the ribosome, oxidative phosphorylation, aminoacyl-tRNA biosynthesis, proteasome, carbon metabolism, and metabolic pathways, were shown in Fig 9A to 9F. Uncharacterized protein had the highest degree of connection among the proteins in the network, followed by ribosomal_L18_c domain-containing protein, S4 RNA-binding domain-containing protein, ribosomal_L2_C domain-containing protein, and ribosomal_S10 domain-containing protein (S2 Table).

### Overviews of Kac proteins in *S*. *invicta*

**Fig 10:**
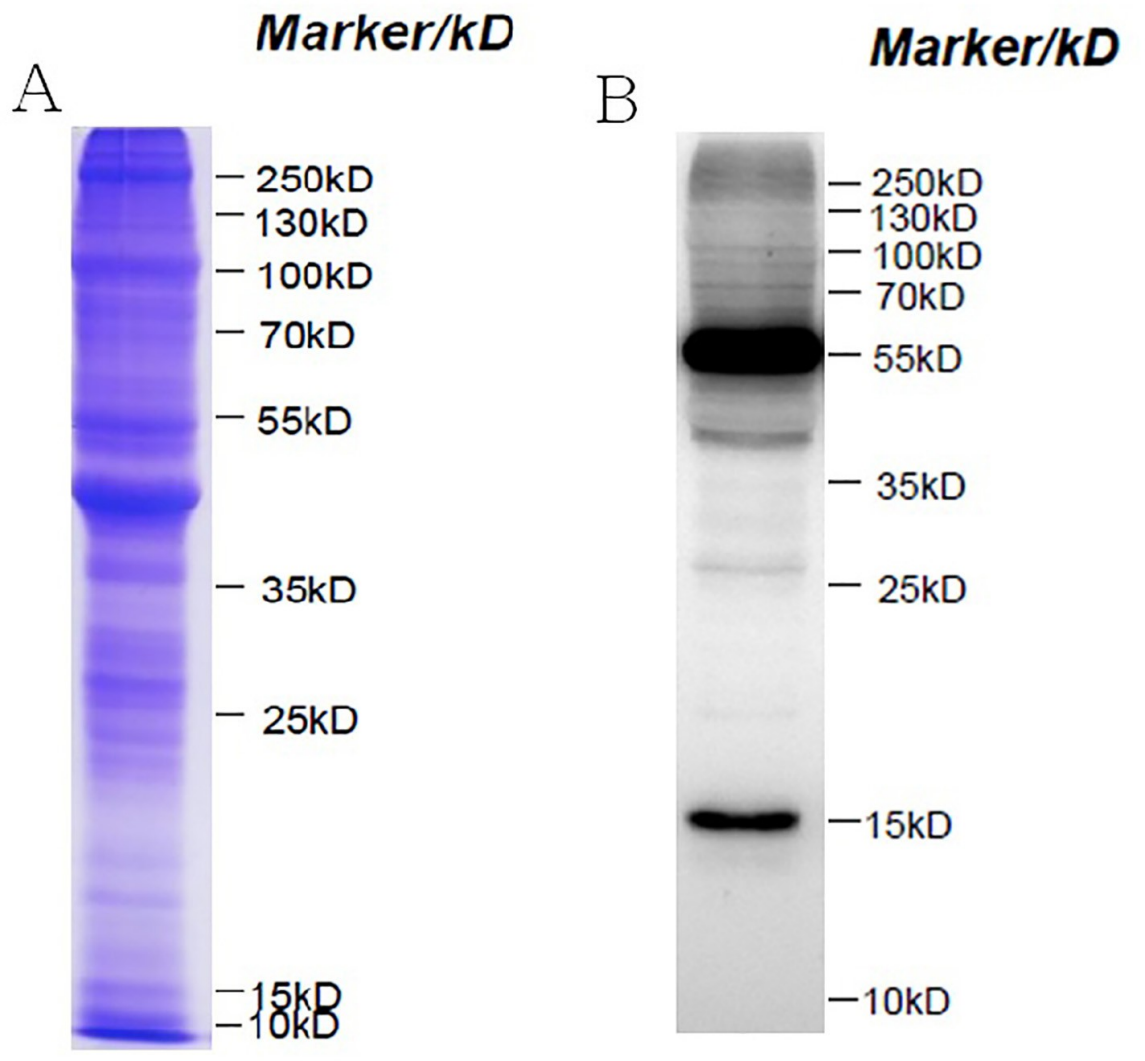
Overviews of lysine acetylation (Kac) proteins in *Solenopsis invicta*. (A) An overview of Kac by SDS-PAGE analysis. (B) An overview of Kac by Western blot analysis. 20 μg protein/lane; Primary antibody: Anti-acetyllysine Antibody (PTM-101: 22838591 HB 14; 1:1000 dilution); 2^nd^ antibody: Thermo, Pierce, Goat anti-Mouse IgG, (H+L), Peroxidase Conjugated, 31430, 1: 5000 dilution.

Western blotting was carried out to supply an overview of Kac in *S*. *invicta* after SDS-PAGE analysis (Fig 10A and 10B). Western blotting with short exposure and long exposure was showed in S1 Fig. Numerous protein bands with a broad extent of molecular weights, including histone and nonhistone proteins, were surveyed (Fig 10B).

## Discussion

Our proteomic analyses of the Hymenoptera insect *S*. *invicta*, the first ever conducting, found several Kac proteins. The results indicated that Kac may play an important role in the sensitization, cellular energy metabolism, immune response, nerve signal transduction, and response to biotic and abiotic stress of *S*. *invicta*.

The distribution of mass errors showed that the exactitude of the modified peptide data gained from MS was great. The length of all Kac peptides was in accordance with the digestive properties of trypsin. Our results identified 2387 acetylation sites in 992 proteins. Nie et al. [9] demonstrated that *B*. *mori* contained 667 Kac sites in 342 acetylated proteins, which was the first time that Kac proteins were identified in this kind of Lepidoptera insect. Weinert et al. [12] identified 1981 Kac sites in the proteome of *D*. *melanogaster*. These suggested that the acetylation level of proteins varied in diverse species.

GO classification demonstrated that acetylated proteins were related to a wide range of biological processes, cellular component as well as molecular function in *S*. *invicta*. Homologous proteins, such as phosphoglycerate kinase, fructose–bisphosphate aldolase, malate dehydrogenase, and citrate synthase, in both human beings and *Drosophila*, were Kac proteins, suggesting a conservation of evolution and function [16–18]. In our study, these proteins were also found to be lysine acetylated, which further proved their conservation. In this study, there were enrichments of KH, KF, KY, KW, KG, and KT, indicating that acetylation tended to take place in protein regions that were rich in lysine. KY or KH motifs also appeared in *B*. *mori*, *D*. *melanogaster*, *Escherichia coli*, and *Streptomyces roseosporus*, which indicated that aromatic, alkaline, and positively charged amino acids may play a key role in acetylation [9]. These also revealed the conservation of acetylated sites between *S*. *invicta* and other species. The distribution of amino acid from positions –6 to +6 that flank Kac sites of *B*. *mori* was highly biased [9], but there was little bias around the amino acid residues in the acetylomes of *D*. *melanogaster* [12]. There was no bias in amino acid distribution in this study.

Most of the identified proteins were localized in the cytoplasm for the predictions of subcellular distribution in this study. These were consistent with that of GO enrichment of biological processes, which demonstrated that most Kac proteins were related to cytoplasmic translation. Many proteins were located in the mitochondria. Acetylated proteins in *B*. *mori* were distributed mainly in cytosol, extracellular space, nucleus, and mitochondria [9]. Kac was widely present in mitochondria, which was critical for the regulation of cellular energy production, metabolism, and cellular homeostasis [19–21]. Moreover, lots of acetylated proteins were present in extracellular or plasma membrane, possibly to monitor cuticular layer-related proteins [22]. The results showed that a number of categories of cellular component, biological process as well as molecular function were significantly enriched, which indicated that acetylation was a regulatory process for those functions. In addition, venom allergen Sol i 2, Sol i 3, and Sol i 4 were found to be located in the extracellular. There are four major allergic proteins, Sol i 1, Sol i 2, Sol i 3, and Sol i 4, in the protein components of *S*. *invicta* venom [23]. These proteins are powerful triggers for allergic reaction [23]. Sol i 2 is the major protein component of the venom and has no cross reaction with other Hymenoptera venoms [24]. Sol i 3 is a member of the antigen 5/pathogenesis-related protein family [23, 25]. Sol i 4, which contains 8% to 10% of the most basic protein components, shares a 37% sequence identity with Sol i 2, but there is no cross reaction in immunity [25]. Sol i 1, Sol i 2 and Sol i 3 are common to fire ants, while Sol i 4 is unique to *S*. *invicta* [24]. The results indicated that Kac may play an important role in the sensitization of *S*. *invicta*.

Acetylated proteins related to the oxidoreductase activity of molecular function were significantly enriched both in *S*. *invicta* and *B*. *mori* [9]. Acetylated protein enrichments associated with several pathways, including translation, transcription, and metabolism, had been reported in diverse eukaryotes and prokaryotes, proving the crucial role of Kac in multiple organisms [22, 26, 27]. Pathway enrichment analyses demonstrated that Kac proteins were involved in various metabolic processes, suggesting that acetylation was vital for adjusting metabolism in *S*. *invicta*. Kac trended to target substance and energy metabolism in *B*. *mori* [9]. This was consistent with our results. Kac proteins involved in the TCA cycle were significantly enriched in our study, as has been found for *B*. *mori* [9]. Interestingly, many Kac proteins were related to arginine and proline metabolism containing proline dehydrogenase. Proline-rich peptides are grouped with antimicrobial peptides, which were significant effectors of the innate immune system in insects [28]. Arginine kinase, which plays an important role in maintaining constant ATP level and in the interrelation between energy production and utilization, is a phosphagen kinase participated in cell metabolism [29, 30]. Wang et al [29] demonstrated that high expression and activity of arginine kinase gene was identified in workers of *S*. *invicta*, because workers developed defensive and sensory organs and functions to defend, forage and maintain colonies. Arginine kinase is not only involved in the energy metabolism and development of insects, but also involved in their immune response [31]. The content of arginine kinase in resistant larvae of *B*. *mori* was significantly higher than that in susceptible larvae [32], indicating that arginine kinase participated in the immune response of *B*. *mori* larvae [31]. Sequence alignment analysis demonstrated that arginine kinase was evolutionarily conserved among different ant species. These indicated that the acetylation of arginine kinase may be involved in the cellular energy metabolism and immune response of *S*. *invicta*. In addition, several Kac proteins were involved in phagosome. Phagosome kills microorganism by promoting the formation of hydrogen peroxide and superoxide [33]. In the process of nerve signal transduction, V-ATPase is related to the accumulation of neurotransmitters in synaptic vesicles [34]. Tubulin sequences are conserved in vertebrates and plants [35]. Tubulin plays an important biological role in response to biotic and abiotic stress [36]. Our results demonstrated that V-type proton ATPase subunit G and tubulin alpha chain were evolutionarily conserved among different ant species. These indicated that the acetylation of V-type proton ATPase subunit G may be involved in the nerve signal transduction of *S*. *invicta*, while the acetylation of tubulin alpha chain may be involved in response to biotic and abiotic stress of *S*. *invicta*. However, these need to be further verified.

Domain-enrichment analyses showed that 25 protein domains, including the pyridine nucleotide-disulphideoxido reductase, tRNA synthetase class II core domain (G, H, P, S, and T), and Acyl-CoA dehydrogenase, were revealed to be significantly enriched substrates. Acyl-CoA dehydrogenase proteins were critical proteins associated with lipid metabolism and transport [37]. Furthermore, our results demonstrated that mitochondrial, nuclear, and cytoplasmic processes may be strictly adjusted by acetylation. Protein interactions played an important role in many biological pathways and monitored almost all cellular processes [38]. 220 acetylated proteins were constructed in a PPI network. The six most interconnected interaction clusters included ribosome, oxidative phosphorylation, aminoacyl-tRNA biosynthesis, proteasome, carbon metabolism, and metabolic pathways. The results suggested that acetylated proteins were associated with a variety of protein interactions, which controlled various signaling pathways in *S*. *invicta*.

Our study of lysine acetylome supplies abundant information for function analysis of reversible Kac in the growth and development of *S*. *invicta* and other Hymenoptera insects. It may be useful to confirm the functions of Kac target proteins for the design of specific and effective drugs to prevent and control this dangerous invasive species. For example, since arginine kinase does not exist in mammalian tissues, it provides a potential target for the development of new pest-specific insecticides against *S*. *invicta* in the future.

## Supporting information

S1 Table Lysine acetylation proteins of *Solenopsis invicta* containing different domains. (XLSX)

S2 Table Lysine acetylation proteins of *Solenopsis invicta* with different degrees of protein–protein interaction. (XLSX)

S1 Fig An overview of lysine acetylation by Western blotting. **(A)** Short exposure (15s). **(B)** Long exposure (30s).
(TIF)

S1 Dataset (XLSX)

S1 Raw images (PDF)
