# Supplementary figures and images for: First proteomic analysis of the role of lysine acetylation in extensive functions in Solenopsis invicta
Source: PLoS One. 2020 Dec 16;15(12):e0243787. doi: 10.1371/journal.pone.0243787 (PMC7743978; doi:10.1371/journal.pone.0243787)

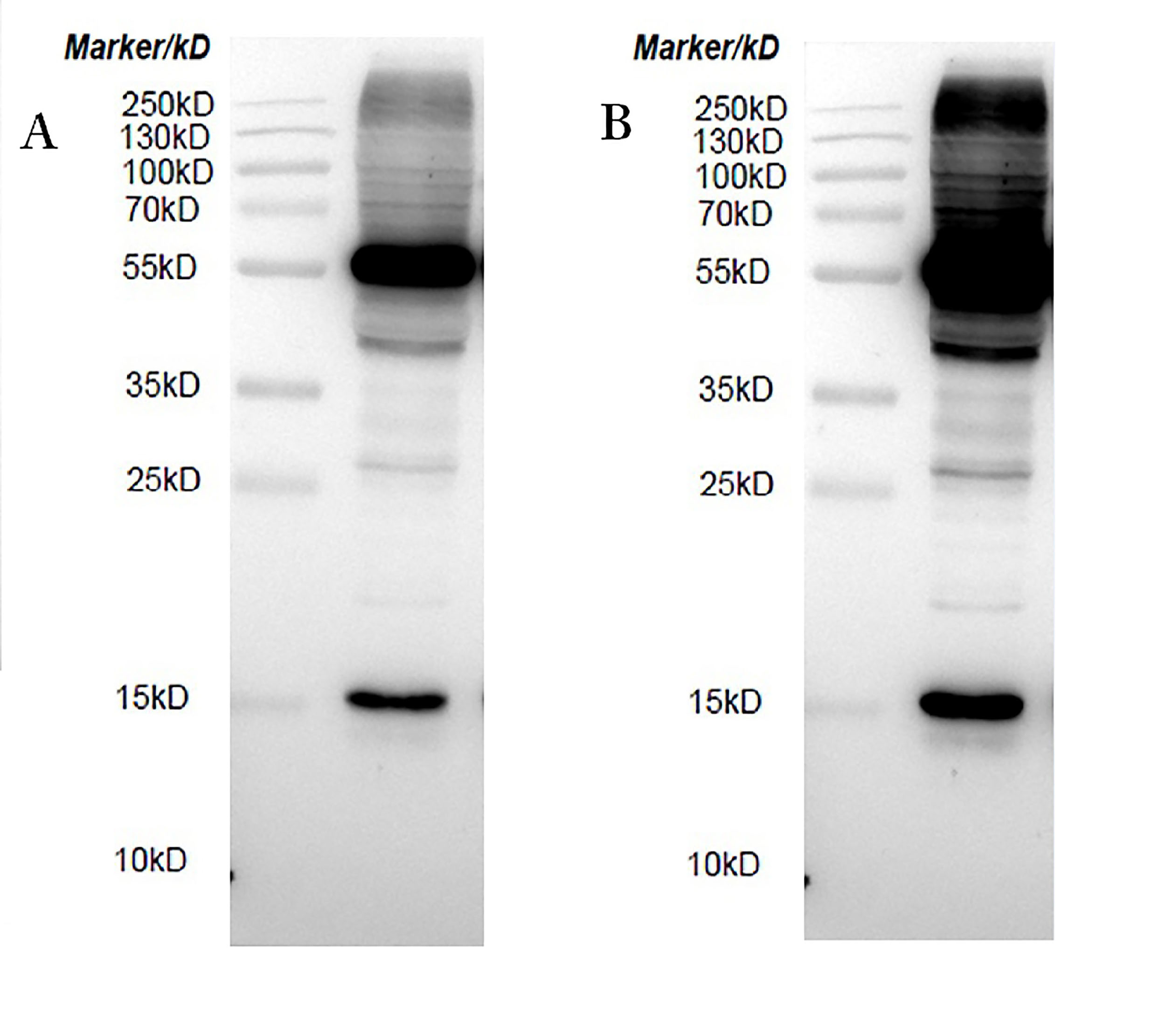

Supplement: S1 Fig — (A) Short exposure (15s). (B) Long exposure (30s). (TIF) [file pone.0243787.s003.tif]
