# Supplementary material for: First proteomic analysis of the role of lysine acetylation in extensive functions in Solenopsis invicta
Source: PLoS One. 2020 Dec 16;15(12):e0243787. doi: 10.1371/journal.pone.0243787 (PMC7743978; doi:10.1371/journal.pone.0243787)
Supplement: S1 Raw images — (PDF) [file pone.0243787.s005.pdf]

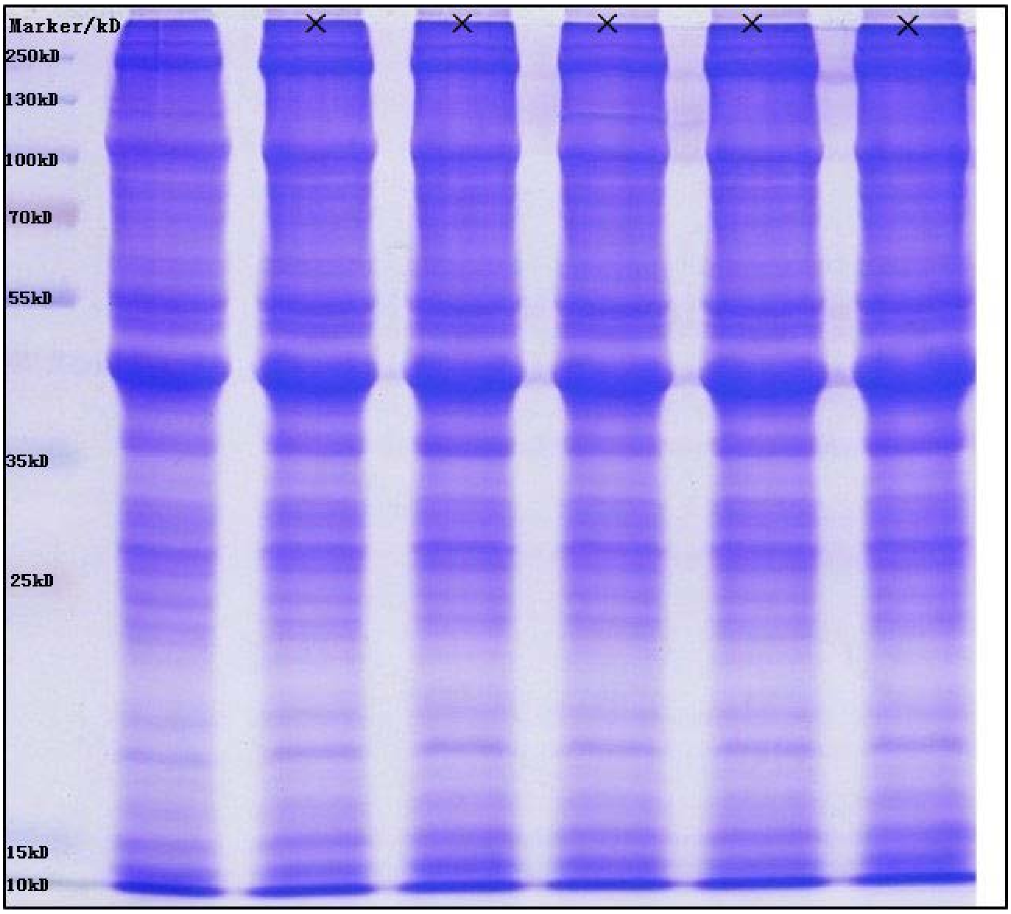

Fig 10A. An overview of lysine acetylation by SDS-PAGE analysis

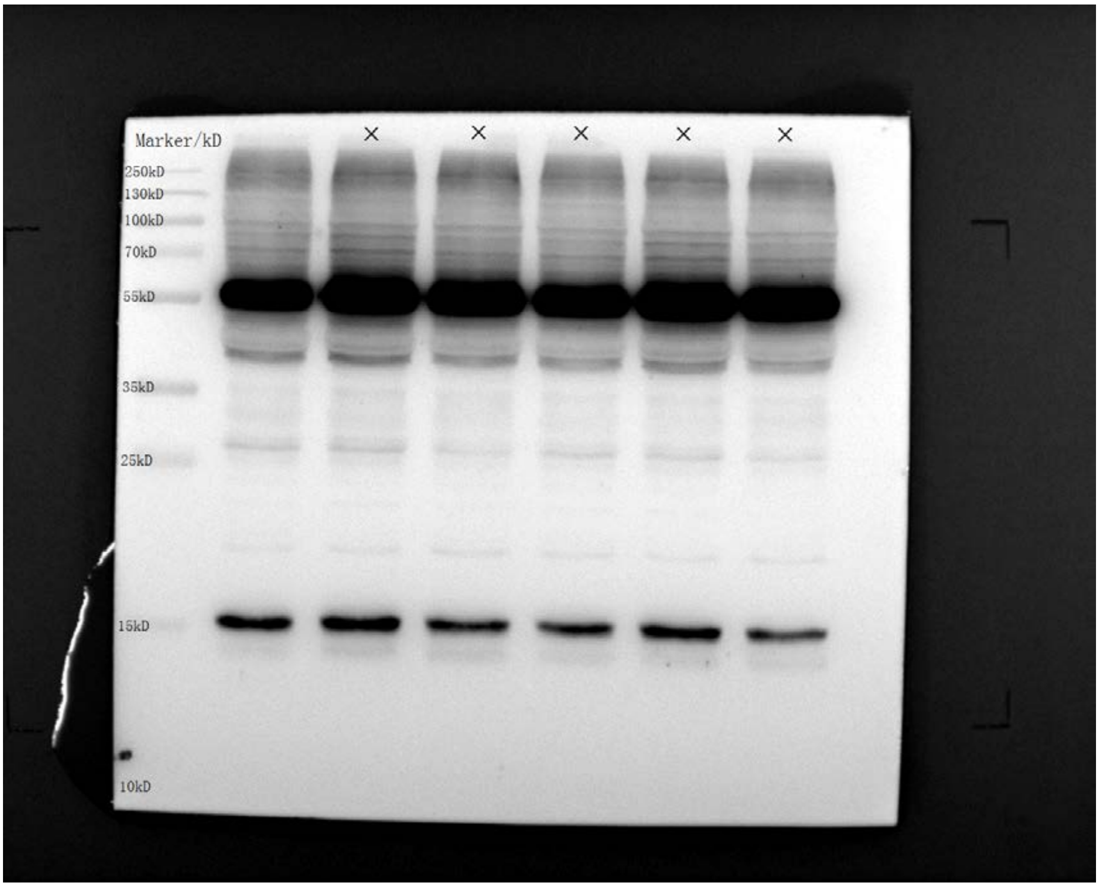

Fig 10B. An overview of lysine acetylation by Western blot analysis.

S1 Figure A. An overview of lysine acetylation by Western blotting: short exposure (15s). 20 μg protein/lane; Primary antibody: Anti-acetyllysine Antibody (PTM-101: 22838591 HB 14; 1:1000 dilution); 2nd antibody: Thermo, Pierce, Goat anti-Mouse IgG, (H+L), Peroxidase Conjugated, 31430, 1: 5000 dilution.

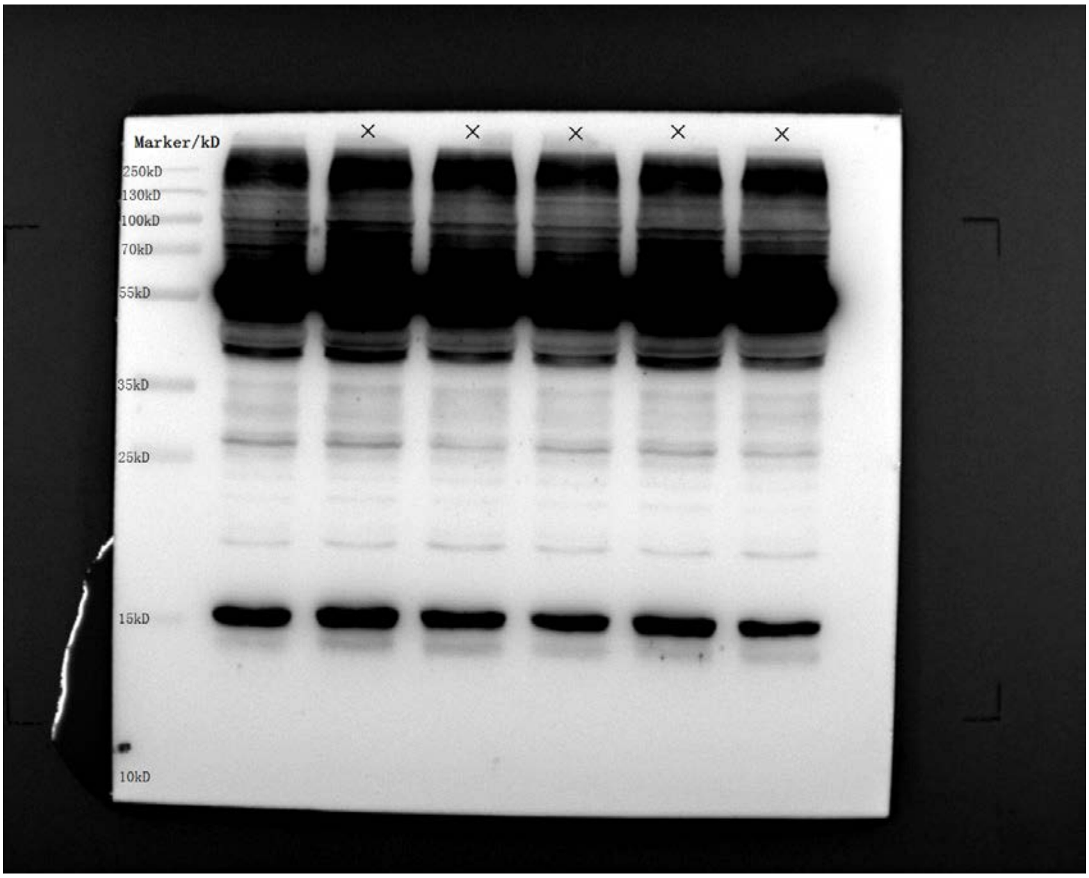

S1 Figure B. An overview of lysine acetylation by Western blotting: long exposure (30s). 20 μg protein/lane; Primary antibody: Anti-acetyllysine Antibody (PTM-101: 22838591 HB 14; 1:1000 dilution); 2nd antibody: Thermo, Pierce, Goat anti-Mouse IgG, (H+L), Peroxidase Conjugated, 31430, 1: 5000 dilution.
